# Editorial: Systemic Coordination of Invertebrate Homeostasis

**DOI:** 10.3389/fphys.2021.736185

**Published:** 2021-09-07

**Authors:** José Luis Ramirez, Isabela Ramos, Fabio M. Gomes

**Affiliations:** ^1^Crop Bioprotection Research Unit, United States Department of Agriculture, National Center for Agricultural Utilization Research, Agricultural Research Service, Peoria, IL, United States; ^2^Instituto de Bioquímica Médica Leopoldo de Meis, Universidade Federal do Rio de Janeiro, Rio de Janeiro, Brazil; ^3^Instituto de Biofísica Carlos Chagas Filho, Universidade Federal do Rio de Janeiro, Rio de Janeiro, Brazil

**Keywords:** homeostasis, microbiome, immunity, hormonal regulation, lipid transport, oogenesis, blood digestion

Multicellularity has allowed highly specialized cells to organize in tissues and organs in order to develop specific functions. While this resulted in an unsurpassable level of efficiency, it became necessary for organs and cells to coordinate their metabolism. In this Research Topic, several new contributing articles provide much-needed new information in this area of research. Overall, these articles highlight how invertebrate organisms have found solutions that allowed them to integrate their physiology to achieve a highly complex systemic coordination of homeostasis.

## The Coordination of Nutrient Uptake and Egg Formation

While several metabolites can be synthesized *de novo* by anabolic reactions, some others can only be obtained through feeding or as by-products from their microbiota's metabolism. To sustain their development, invertebrates must not only obtain a proper level of nutrients; but also have to metabolize and coordinate their storage and distribution to remaining organs (Karasov and Douglas, 2013). In this Research Topic, Entringer et al. studied the fate of cholesterol in the kissing bug *Rhodnius prolixus*. Unlike mammals, insects are not able to convert acetate into cholesterol. The authors investigated the coordination between cholesterol intake and storage by different organs (Entringer et al.). Their results showed that multiple gut epithelia segments can absorb cholesterol, which transiently accumulates in the fat body, with very small levels of cholesterol being found available in the hemolymph. They have also observed that nutrients are later used for the maturation of ovaries and the build-up of vitellogenic oocytes, where cholesterol progressively accumulates.

The coordination of such events must rely on signaling molecules that coordinate interorgan and cell-cell communication. For example, insulin-like peptides (ILPs—analogous to vertebrate insulin) and their counterpart adipokinetic hormone (AKH—analogous to vertebrate glucagon) have a major role in regulating nutrient homeostasis by coordinating nutrient-sensing pathways, such as the PI3K-AKT-TOR pathways (Nässel and Vanden Broeck, 2016). Also working with *R. prolixus*, Leyria et al. showed that trehalose stores follow a pattern of accumulation during vitellogenesis due to the transcription of the trehalose-specific facilitated transporter gene (Rhopr-TRET) in the ovaries. The authors also showed that ILPs and the adipokinetic hormone (AKH) mediate Rhopr-TRET expression, providing a link between blood-feeding, insulin signaling, and trehalose accumulation and mobilization during vitellogenesis.

Choriogenesis is the last stage of oocyte maturation before fertilization. At this stage, the follicle cells assemble the chorion layers, which will allow gas exchange and provide protection, while impairing desiccation for the developing embryo. Follicle cells can coordinate the assembly of a set of proteins generating an intricate network of macromolecules on the chorion. However, the exact mechanisms by which this is accomplished are only partially understood. Santos and Ramos studied *R. prolixus* choriogenesis and provided evidence that ATG3 (autophagy-related gene 3) is highly expressed in the ovaries during vitellogenesis. They further showed that parental deficiency in ATG3 impairs autophagosome formation and proper chorion biogenesis by the follicle cells, resulting in altered chorion ultrastructure and protein composition. Further studies will allow a finer understanding of the triggering signals and the signaling pathways that regulate ATG3 function during choriogenesis.

## Interorgan Communication of Tissue Homeostasis

Coordination of physiology and behavior is highly dependent on interorgan communication, which is provided for the most part by hormonal regulation. In insects, the molting hormone 20-hydroxyecdysone (20E) is an ecdysteroid derived from cholesterol metabolism. During larval development, 20E is synthesized in the prothoracic gland and coordinates insect molt by interacting with the ecdysteroid receptor, a heterodimer composed by ecdysone receptor (EcR) and ultraspiracle (USP) (Yao et al., 1993). Shi et al. used video recording to identify the phases of cocoon-spinning in the diamondback moth *Plutella xylostella*. The authors identified three successive phases (selection of a pupation site, spinning a loose cocoon, and padding the scaffold cocoon). Furthermore, they demonstrated that 20E plays an important role in cocoon-spinning behavior via the modulation of silk gland fibroin genes.

Apart from lipid hormones, several other components participate in systemic coordination of invertebrate physiology. Wound healing is a key aspect of tissue homeostasis. During this process, cells must be driven to the site of injury in order to rapidly repair the injured tissue and prevent pathogen invasion. In the leech *Hirudo verbana*, the T2 ribonuclease HvRNASET2 (rHvRNASET2) stimulates the production of collagen and is involved in the extracellular matrix remodeling, by acting as chemoattractants for macrophages (Baranzini et al., 2019). Through a series of morphological studies coupled with molecular bioassays, Baranzini et al. demonstrated the remarkable complexity of tissue homeostasis maintenance during the wound healing process. Leeches injected with rHvRNASET2 induced macrophage recruitment, collagen synthesis, and participated in muscle tissue regeneration. The authors also described the homeostatic role of rHvRNASET2 in vasculogenesis and angiogenesis during the recruitment of myoendothelial vessel-associated precursor cells.

## The Coordination of Hematophagy and Vector Competence

In hematophagous insects, blood is a key source of nutrients for egg production. Gandara et al. studied the role of NADPH oxidases and xanthine dehydrogenases (XDH) in *R. prolixus* digestive physiology. They show that superoxide produced by NADPH oxidase 5 (NOX5) and urate produced by XDH coordinate midgut peristalsis and are key to blood digestion. Blood ingestion is correlated with an increase in midgut microbial load, which facilitates blood digestion and regulates host immunological status (Oliveira et al., 2011). As many species developed a preference for human blood, hematophagy also allowed several invertebrate species to act as vectors of human diseases. Talyuli et al. presented a thorough review of how blood-feeding triggers several aspects of vector competence that are not canonical components of the invertebrate immune system.

Indeed, it has now become clear that tissue homeostasis and vector immunity coordinate invertebrate responses to infections. Redox homeostasis during blood digestion is a key component of this coordination (Talyuli et al.). During *Plasmodium* invasion of the midgut of *Anopheles gambiae*, the activation of nitric oxide synthase (NOS) drives the accumulation of nitric oxide (NO). As a result, reactive oxygen species (ROS) detoxifying enzymes, such as heme-peroxidases are produced under the control of c-Jun N-terminal kinases (JNK) signaling and ultimately nitrify the parasite, targeting it for killing by mosquito humoral factors (Oliveira et al., 2012). In this Research Topic, Kakani et al. provided evidence of an additional role for heme-peroxidase 2 in the Asian malaria vector *Anopheles stephensi* in regulating the gut microbiota. They propose that enterocyte-produced heme-peroxidase 2 (AsHPX2) regulates the levels of ROS at the gut lumen and that a reduction of AsHPX2 following blood-feeding creates proper physicochemical conditions for the expansion of the gut microbiota, which is essential for blood digestion.

## The Interplay Between Host and Host Microbiota Regulating Vector Competence

Manipulation of microbiota is a promising strategy to manipulate vector competence and diminish transmission of vector-borne pathogens. For example, the release of *Wolbachia*-infected mosquitoes has become one of the most promising strategies for arbovirus control. Upon *Wolbachia* infection, natural populations of *Aedes aegypti* become refractory to dengue (DENV) and Zika (ZIVK) viruses. More recently, field studies using this approach have shown a strong effect in the reduction of dengue fever cases (Utarini et al., 2021). However, the molecular mechanisms underlying *Wolbachia-*Aedes-arbovirus regulation of vector competence remain uncertain. Martins et al. performed quantitative mass spectrometry-based proteomics in sections of heads and salivary glands of ZIKV and *Wolbachia*-infected *Ae. aegypti*. Their study provides a panel of proteins and pathways belonging to diverse biological processes and physiological pathways that are under modulation by Zika and *Wolbachia* including the *Wolbachia*-induced effects on ROS production and the modulation of immune pathways. Such datasets can provide invaluable data for the refinement of the interaction models between *Wolbachia* and *Aedes* in future studies.

## Author Contributions

JR and IR contributed to the idealization, development of the Research Topics and editorial, and reviewed this manuscript. FG contributed to the idealization, development of the Research Topics and editorial, and prepared the draft and final version of this manuscript. All authors contributed to the article and approved the submitted version.

## Funding

This work was supported by the Serrapilheira Institute (Grant number R-1912-32058).

## Conflict of Interest

The authors declare that the research was conducted in the absence of any commercial or financial relationships that could be construed as a potential conflict of interest.

## Publisher's Note

All claims expressed in this article are solely those of the authors and do not necessarily represent those of their affiliated organizations, or those of the publisher, the editors and the reviewers. Any product that may be evaluated in this article, or claim that may be made by its manufacturer, is not guaranteed or endorsed by the publisher.
